# Crystal structure of 2-[(naphthalen-2-yl)meth­yl]iso­thio­uronium bromide

**DOI:** 10.1107/S2414314620015114

**Published:** 2020-11-17

**Authors:** Václav Eigner

**Affiliations:** a Institute of Physics AS CR, v.v.i., Na Slovance 2, 182 21 Prague 8, Czech Republic; University of Otago, New Zealand

**Keywords:** crystal structure, iso­thio­uronium salt, hydrogen bonds, dipole–dipole inter­actions

## Abstract

Herein we report the crystal structure of 2-[(naphthalen-2-yl)meth­yl]iso­thio­uronium bromide, C_12_H_13_N_2_S^+^·Br^−^, which crystallizes in the monoclinic *P*2_1_/*c* centrosymmetric space group. The asymmetric unit contains one 2-[(naphthalen-2-yl)meth­yl]iso­thio­uronium cation and one bromide anion. The methyl­ene carbon lies in plane of the naphthalene core. In comparison with reference structures, elongation of C—S bonds as well as tilting of the iso­thio­uronium group is observed. Given the ionic nature of the compound, the structure is held by charge-assisted N—H⋯Br hydrogen bonds, with a noteworthy contribution of dipole–dipole inter­actions, which form bilayers in the structure. The bilayers are held by the weak London forces.

## Abstract 2

The crystal structure of the title salt is held by N—H⋯Br charge-assisted hydrogen bonds and dipole–dipole inter­actions. In comparison with reference structures, elongation of C—S bonds is observed.

## Graphical abstract

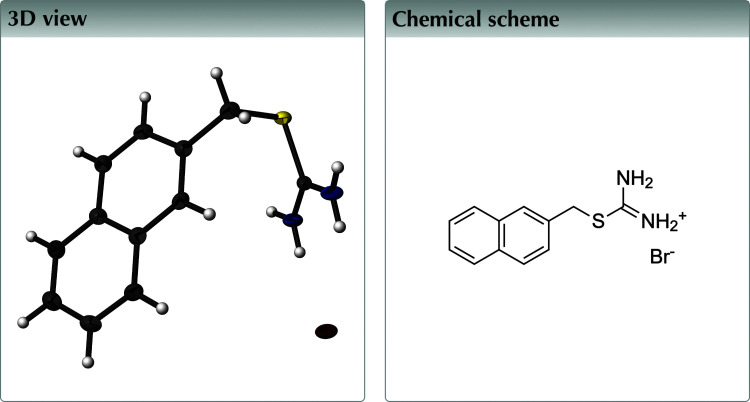

## Structure description

Iso­thio­uronium salts have been investigated because of their ability to bind anions by charge-assisted hydrogen bonds (Yeo & Hong, 1998 ▸; Seong *et al.*, 2004 ▸). Despite their potential in crystal engineering, only 23 crystal structures of 2-(aryl­meth­yl)iso­thio­uronium salts are present in the CSD (Groom *et al.*, 2016 ▸). In our studies of iso­thio­uronia, we have managed to synthesize and crystallize naphthalene-2-ylmethyl-bearing iso­thio­uronium bromide, and we report here its structure and a comparison with similar crystal structures.

**Figure 1:**
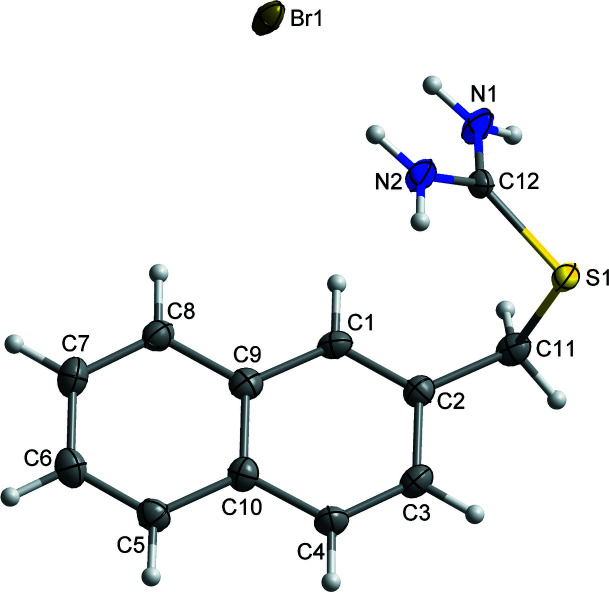
The title compound showing the numbering scheme with displacement ellipsoids drawn at the 50% probability level. H atoms are shown as spheres of arbitrary radius.

The title compound crystallizes in the monoclinic *P*2_1_/*c* centrosymmetric space group with one 2-[(naphthalen-2-yl)meth­yl]iso­thio­uronium cation and one bromide anion in the asymmetric unit, see Fig. 1 ▸. The naphthalene core is almost perfectly planar, with a maximum deviation of 0.026 (3) Å for atom C6. The C11 atom can be considered to be in the plane of naphthalene core, with a deviation of 0.039 (3) Å from the mean plane. The single bonds around carbon C11 and S1 allow for the free rotation of the iso­thio­uronium group.

There are no 2-[(naphthalen-2-yl)meth­yl]iso­thio­uronia structures in the the CSD; therefore, we decided to compare the title compound with 2-benzyl­iso­thio­uronia structures. The published structures of 2-benzyl­iso­thio­uronia can be divided according to the C_ar_—C_me_—S—C_th_ torsion angle into linear and non-linear groups. Since the relevant torsion angle of the title compound is −68.1 (3)°, only the non-linear 2-benzyl­iso­thio­uronia, will be used as a reference group [CCDC codes EBIFOK (Ishii *et al.*, 2000 ▸), IGECIG (Raptopoulou *et al.*, 2002 ▸), JALSOE (Mikolajczyk *et al.*, 1989 ▸), SEFRUQ (Barker & Powell, 1998 ▸), TAWVAP (Stergiou *et al.*, 2005 ▸), TOBNEE (Gayathri *et al.*, 2008 ▸) and YOCRUE (Fun *et al.*, 2008 ▸)]. The C2—C11 bond length of 1.495 (5) Å in the title compound is within the usually observed values among the reference group, while the C11—S1 and S1—C12 bond lengths of 1.855 (3) Å and 1.771 (3) Å respectively are larger than usually observed, with average values of 1.819 Å and 1.739 Å. The C2—C11—S1 and C11—S1—C12 angles of 111.7 (2) and 96.98 (15)°, respectively, are less obtuse than the average values among the reference group, 115.21 and 103.91°. This deviation is most likely caused by the difference of electronic behaviour between the benzyl group and the 2-naphthyl­methyl unit.

The C11—S1 bond is almost perpendicular to the naphthalene core, with C1—C2—C11—S1 and C3—C2—C11—S1 torsion angle values of 91.8 (3) and −88.0 (3)°, respectively. Among the reference group, such a conformation is unusual, with average values being 61.45 and 120.53°. The iso­thio­uronium group is significantly tilted, with C11—S1—C12—N1 and C11—S1—C12—N2 torsion angles of −68.5 (3) and 110.7 (3)°, respectively. Such a tilt is not observed among the reference group, where the average values are 20.46 and 160.94°. The tilting of the group can be explained by the steric demands of the 2-naphthyl­methyl unit on the packing.

**Figure 2:**
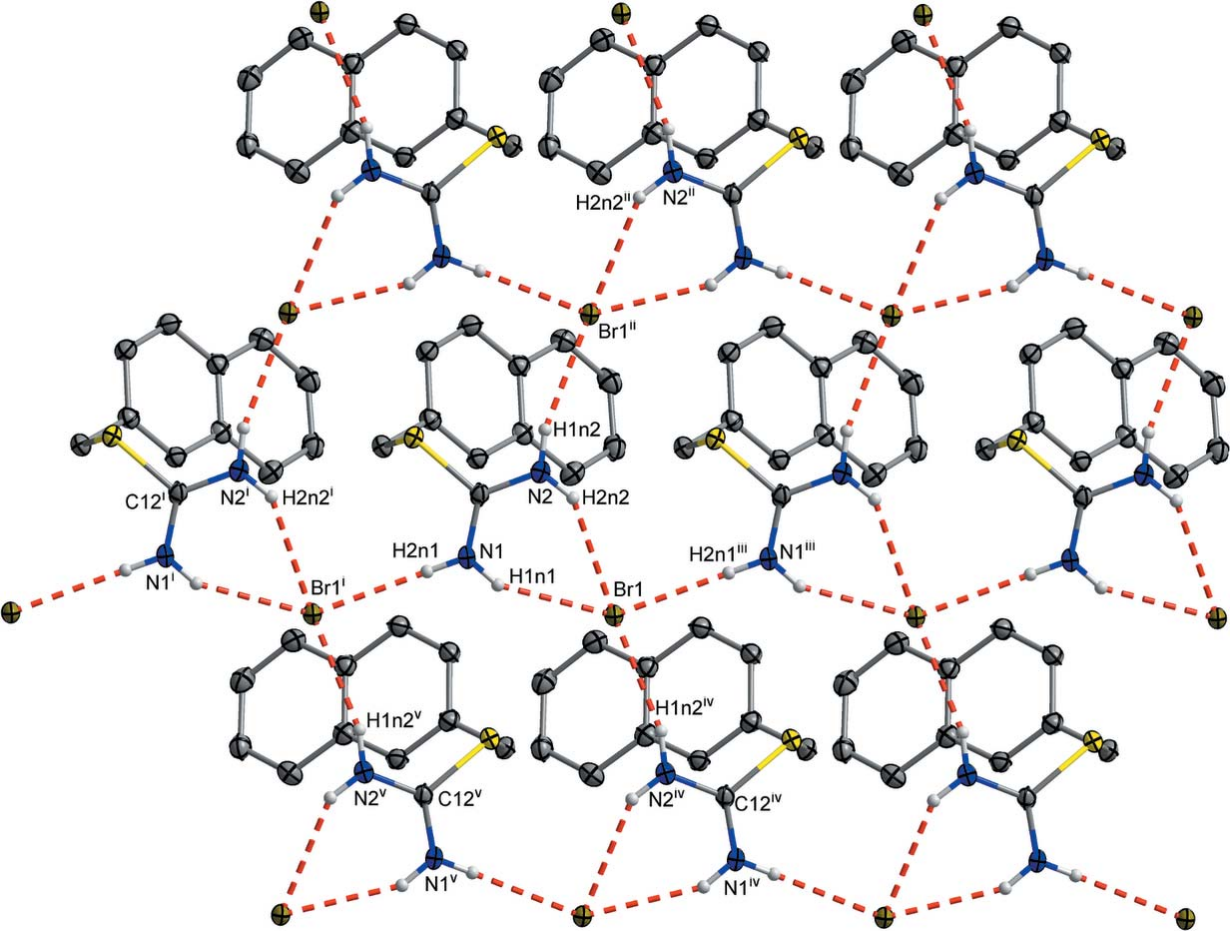
The charge-assisted hydrogen bonds in the title compound. Hydrogen atoms not involved in hydrogen bonding are omitted for clarity. Symmetry codes: (i) *x*, *y* − 1, *z*; (ii) *x*, −*y* + 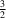 , *z* − 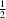 ; (iii) *x*, *y* + 1, *z*; (iv) *x*, −*y* + 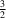 , *z* + 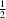 ; (v) *x*, −*y* + 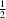 , *z* + 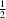 .

**Figure 3:**
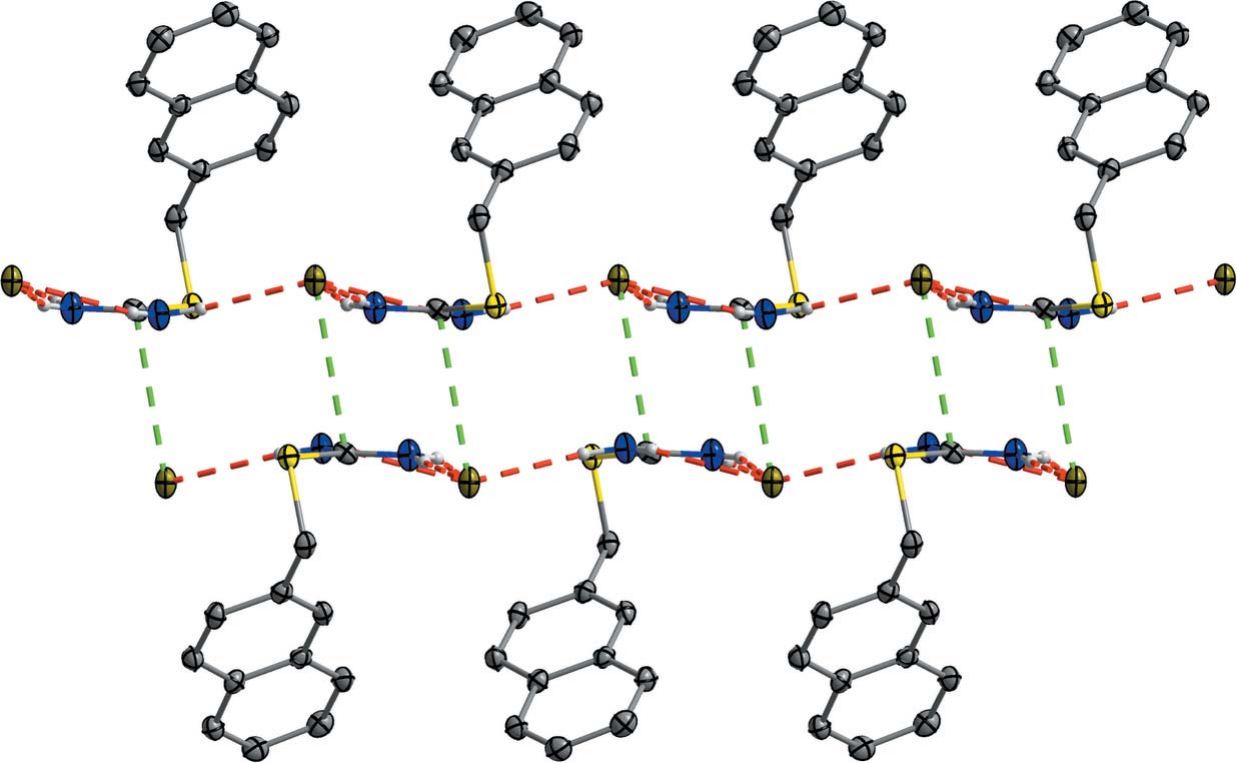
The bilayers formed in the title compound by charge-assisted hydrogen bonds, depicted in red, and dipole–dipole inter­actions, depicted in green. Hydrogen atoms not involved in hydrogen bonding are omitted for clarity.

The N—H⋯Br charge-assisted hydrogen bonds (Table 1 ▸) have the most significant impact on crystal structure of 2-[(naphthalen-2-yl)meth­yl]iso­thio­uronium bromide, with every iso­thio­uronium cation forming three hydrogen bonds, one of them bifurcated, with bromide anions. This is a major difference from the structure of 2-benzyl­iso­thio­uronium chloride (Barker & Powell, 1998 ▸), the only structure of 2-aryl­methyl­iso­thio­uronium with simple halide anion, where the iso­thio­uronium group forms four charge-assisted hydrogen bonds with four different chloride anions. The charge-assisted hydrogen bonds form layers in the structure through five chains, N1—H1*n*1⋯Br1⋯H2*n*1^iii^—N1^iii^, and N2—H1*n*2⋯Br1^ii^⋯H2*n*2^ii^—N2^ii^ classified as 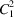 (4) and N1—H1*n*1⋯Br1⋯H1*n*2^iv^—N2^iv^—C12^iv^—N1^iv^, N1—H2*n*1⋯Br1^i^⋯H1*n*2^v^—N2^v^—C12^v^—N1^v^, and N1—H2*n*1⋯Br1^i^⋯H2*n*2^i^—N2^i^—C12^i^—N1^i^ classified as 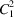 (6) [symmetry codes: (i) *x*, *y* − 1, *z*; (ii) *x*, −*y* + 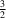 , *z* − 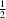 ; (iii) *x*, *y* + 1, *z*; (iv) *x*, −*y* + 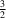 , *z* + 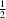 ; (v) *x*, −*y* + 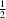 , *z* + 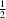 ], see Fig. 2 ▸. The layers are connected by dipole–dipole inter­actions between C12 and Br1^vi^ [symmetry code: (vi) −*x*, *y* − 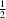 , −*z* + 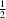 ] with a distance of 3.535 (4) Å into bilayers along the (100) plane, see Fig. 3 ▸. The bilayers are held by weak London forces only.

## Synthesis and crystallization

Thio­urea (0.23 g, 3 mmol) was dissolved in 25 ml of anhydrous aceto­nitrile. The solution was then treated with 2-(bromo­meth­yl)naphthalene (0.55 g, 2.5 mmol). The reaction mixture was stirred for 4 h at room temperature. The resulting white precipitate was filtered and washed with diethyl ether and left to dry at room temperature, resulting in yield 0.72 g (97%) of 2-[(naphthalen-2-yl)meth­yl]iso­thio­uronium bromide.

The 2-[(naphthalen-2-yl)meth­yl]iso­thio­uronium bromide (20 mg) was dissolved in 10 ml of methanol and left to slowly evaporate at room temperature. After 5 d, colorless platelets were collected.

## Refinement

Crystal data, data collection and structure refinement details are summarized in Table 2 ▸.

## Supplementary Material

Crystal structure: contains datablock(s) global, I. DOI: 10.1107/S2414314620015114/sj4217sup1.cif

Structure factors: contains datablock(s) I. DOI: 10.1107/S2414314620015114/sj4217Isup2.hkl

Supporting information file. DOI: 10.1107/S2414314620015114/sj4217Isup3.mol

CCDC reference: 2044275

Additional supporting information:  crystallographic information; 3D view; checkCIF report

## Figures and Tables

**Table 1:** Hydrogen-bond geometry (Å, °)

*D*—H⋯*A*	*D*—H	H⋯*A*	*D*⋯*A*	*D*—H⋯*A*
N1—H1*n*1⋯Br1	0.86 (3)	2.54 (3)	3.332 (3)	153 (3)
N1—H2*n*1⋯Br1^i^	0.86 (2)	2.49 (2)	3.350 (3)	180 (3)
N2—H1*n*2⋯Br1^ii^	0.860 (19)	2.55 (3)	3.381 (3)	164 (4)
N2—H2*n*2⋯Br1	0.86 (3)	2.68 (3)	3.434 (3)	147 (3)

Symmetry codes: (i) 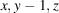 ; (ii) 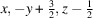 .

**Table 2:** Experimental details

Crystal data	
Chemical formula	C_12_H_13_N_2_S^+^·Br^−^
*M*_r_	297.2
Crystal system, space group	Monoclinic, *P*2_1_/*c*
Temperature (K)	120
*a*, *b*, *c* (Å)	16.2976 (8), 6.1294 (2), 12.2221 (6)
β (°)	99.070 (4)
*V* (Å^3^)	1205.65 (9)
*Z*	4
Radiation type	Cu *K*α
μ (mm^−1^)	6.04
Crystal size (mm)	0.21 × 0.08 × 0.01

Data collection	
Diffractometer	Rigaku Oxford Diffraction Gemini ultra, AtlasS2
Absorption correction	Analytical *CrysAlis PRO* (Rigaku OD, 2015 ▸)
*T*_min_, *T*_max_	0.522, 0.917
No. of measured, independent and observed [*I* > 3σ(*I*)] reflections	12512, 2162, 1699
*R*_int_	0.057
(sin θ/λ)_max_ (Å^−1^)	0.598

Refinement	
*R*[*F* ^2^ > 2σ(*F* ^2^)], *wR*(*F* ^2^), *S*	0.030, 0.081, 1.18
No. of reflections	2162
No. of parameters	157
No. of restraints	4
H-atom treatment	H atoms treated by a mixture of independent and constrained refinement
Δρ_max_, Δρ_min_ (e Å^−3^)	0.48, −0.36

Computer programs: *CrysAlis PRO* (Rigaku OD, 2015 ▸), *SUPERFLIP* (Palatinus & Chapuis, 2007 ▸), *MCE* (Rohlíček & Hušák, 2007 ▸), *DIAMOND* (Brandenburg & Putz, 1999 ▸), *JANA2006* (Petricek *et al.*, 2014 ▸) and *publCIF* (Westrip, 2010 ▸).

## full crystallographic data

### Crystal data

**Table:**

C_12_H_13_N_2_S^+^·Br^−^	*F*(000) = 600
*M**_r_* = 297.2	*D*_x_ = 1.637 Mg m^−^^3^
Monoclinic, *P*2_1_/*c*	Cu *K*α radiation, λ = 1.54184 Å
Hall symbol: -P 2ycb	Cell parameters from 3162 reflections
*a* = 16.2976 (8) Å	θ = 7.4–67.1°
*b* = 6.1294 (2) Å	µ = 6.04 mm^−^^1^
*c* = 12.2221 (6) Å	*T* = 120 K
β = 99.070 (4)°	Platelet, colourless
*V* = 1205.65 (9) Å^3^	0.21 × 0.08 × 0.01 mm
*Z* = 4	

### Data collection

**Table:**

Rigaku Oxford Diffraction Gemini ultra, AtlasS2 diffractometer	2162 independent reflections
Radiation source: X-ray tube	1699 reflections with *I* > 3σ(*I*)
Mirror monochromator	*R*_int_ = 0.057
Detector resolution: 5.1783 pixels mm^-1^	θ_max_ = 67.2°, θ_min_ = 5.5°
ω scans	*h* = −19→18
Absorption correction: analytical CrysAlisPro (Rigaku OD, 2015)	*k* = −7→6
*T*_min_ = 0.522, *T*_max_ = 0.917	*l* = −14→13
12512 measured reflections	

### Refinement

**Table:**

Refinement on *F*^2^	40 constraints
*R*[*F*^2^ > 2σ(*F*^2^)] = 0.030	H atoms treated by a mixture of independent and constrained refinement
*wR*(*F*^2^) = 0.081	Weighting scheme based on measured s.u.'s *w* = 1/(σ^2^(*I*) + 0.0016*I*^2^)
*S* = 1.18	(Δ/σ)_max_ = 0.017
2162 reflections	Δρ_max_ = 0.48 e Å^−^^3^
157 parameters	Δρ_min_ = −0.36 e Å^−^^3^
4 restraints	

### Fractional atomic coordinates and isotropic or equivalent isotropic displacement parameters (Å^2^)

**Table:**

	*x*	*y*	*z*	*U*_iso_*/*U*_eq_	
Br1	0.12634 (2)	0.79627 (6)	0.40347 (3)	0.02124 (12)	
S1	0.09471 (5)	0.11747 (14)	0.09911 (6)	0.0181 (2)	
N1	0.09225 (19)	0.2979 (5)	0.2973 (2)	0.0222 (9)	
N2	0.08363 (19)	0.5365 (5)	0.1520 (2)	0.0221 (9)	
C1	0.2898 (2)	0.3770 (6)	0.1961 (3)	0.0188 (10)	
C2	0.2633 (2)	0.2128 (6)	0.1229 (3)	0.0193 (10)	
C3	0.2926 (2)	0.2072 (6)	0.0196 (3)	0.0211 (10)	
C4	0.3465 (2)	0.3612 (6)	−0.0066 (3)	0.0224 (11)	
C5	0.4332 (2)	0.6914 (6)	0.0454 (3)	0.0222 (10)	
C6	0.4595 (2)	0.8519 (6)	0.1203 (3)	0.0252 (11)	
C7	0.4283 (2)	0.8638 (6)	0.2208 (3)	0.0245 (11)	
C8	0.3725 (2)	0.7122 (6)	0.2466 (3)	0.0222 (10)	
C9	0.3453 (2)	0.5398 (6)	0.1715 (3)	0.0192 (10)	
C10	0.3751 (2)	0.5322 (6)	0.0686 (3)	0.0198 (10)	
C11	0.2043 (2)	0.0410 (6)	0.1493 (3)	0.0208 (10)	
C12	0.0894 (2)	0.3369 (5)	0.1920 (3)	0.0177 (10)	
H1c1	0.270001	0.38133	0.266045	0.0226*	
H1c3	0.27426	0.093362	−0.032466	0.0253*	
H1c4	0.365563	0.354385	−0.076939	0.0269*	
H1c5	0.45425	0.686312	−0.023628	0.0266*	
H1c6	0.499614	0.957074	0.104151	0.0302*	
H1c7	0.44616	0.979455	0.272054	0.0294*	
H1c8	0.351646	0.722293	0.315701	0.0267*	
H1c11	0.211892	0.018125	0.227929	0.0249*	
H2c11	0.216623	−0.09448	0.115986	0.0249*	
H1n1	0.096 (3)	0.403 (5)	0.344 (3)	0.0267*	
H2n1	0.101 (3)	0.169 (3)	0.324 (3)	0.0267*	
H1n2	0.091 (3)	0.552 (7)	0.0843 (11)	0.0266*	
H2n2	0.086 (3)	0.646 (4)	0.196 (3)	0.0266*	

### Atomic displacement parameters (Å^2^)

**Table:**

	*U*^11^	*U*^22^	*U*^33^	*U*^12^	*U*^13^	*U*^23^
Br1	0.0348 (2)	0.01257 (19)	0.01671 (19)	0.00035 (15)	0.00505 (13)	0.00032 (13)
S1	0.0234 (4)	0.0138 (4)	0.0174 (4)	−0.0010 (3)	0.0041 (3)	−0.0024 (3)
N1	0.0367 (16)	0.0108 (14)	0.0198 (15)	0.0000 (13)	0.0066 (12)	0.0011 (12)
N2	0.0337 (16)	0.0142 (15)	0.0193 (14)	−0.0026 (12)	0.0068 (12)	−0.0022 (11)
C1	0.0220 (16)	0.0188 (17)	0.0164 (16)	0.0038 (14)	0.0056 (12)	0.0012 (13)
C2	0.0202 (16)	0.0183 (17)	0.0191 (16)	0.0011 (14)	0.0023 (12)	0.0005 (14)
C3	0.0262 (17)	0.0185 (17)	0.0184 (16)	0.0016 (14)	0.0026 (13)	−0.0028 (14)
C4	0.0249 (17)	0.025 (2)	0.0180 (16)	−0.0020 (14)	0.0058 (13)	−0.0025 (13)
C5	0.0228 (17)	0.0234 (19)	0.0205 (17)	−0.0008 (15)	0.0040 (13)	0.0018 (14)
C6	0.0248 (17)	0.023 (2)	0.0274 (18)	−0.0030 (14)	0.0024 (14)	0.0036 (14)
C7	0.0276 (18)	0.0184 (18)	0.0262 (18)	−0.0023 (14)	0.0001 (14)	−0.0011 (14)
C8	0.0227 (17)	0.0231 (19)	0.0207 (17)	0.0005 (14)	0.0029 (13)	−0.0045 (14)
C9	0.0176 (16)	0.0199 (18)	0.0192 (16)	0.0035 (13)	0.0001 (12)	0.0013 (13)
C10	0.0216 (16)	0.0179 (18)	0.0195 (16)	0.0006 (14)	0.0017 (13)	0.0019 (13)
C11	0.0270 (18)	0.0173 (18)	0.0183 (16)	0.0041 (14)	0.0043 (13)	0.0026 (13)
C12	0.0190 (16)	0.0127 (18)	0.0228 (16)	−0.0014 (12)	0.0077 (12)	−0.0003 (12)

### Geometric parameters (Å, º)

**Table:**

S1—C11	1.855 (3)	C4—C10	1.423 (5)
S1—C12	1.771 (3)	C4—H1c4	0.96
N1—C12	1.302 (4)	C5—C6	1.366 (5)
N1—H1n1	0.86 (3)	C5—C10	1.419 (5)
N1—H2n1	0.86 (2)	C5—H1c5	0.96
N2—C12	1.316 (4)	C6—C7	1.404 (5)
N2—H1n2	0.860 (19)	C6—H1c6	0.96
N2—H2n2	0.86 (3)	C7—C8	1.372 (5)
C1—C2	1.370 (5)	C7—H1c7	0.96
C1—C9	1.411 (5)	C8—C9	1.424 (5)
C1—H1c1	0.96	C8—H1c8	0.96
C2—C3	1.419 (5)	C9—C10	1.419 (5)
C2—C11	1.495 (5)	C11—H1c11	0.96
C3—C4	1.361 (5)	C11—H2c11	0.96
C3—H1c3	0.96		
			
C11—S1—C12	96.98 (15)	C5—C6—H1c6	119.84
C12—N1—H1n1	121 (2)	C7—C6—H1c6	119.84
C12—N1—H2n1	122 (3)	C6—C7—C8	120.7 (3)
H1n1—N1—H2n1	117 (3)	C6—C7—H1c7	119.63
C12—N2—H1n2	117 (3)	C8—C7—H1c7	119.63
C12—N2—H2n2	120 (2)	C7—C8—C9	120.3 (3)
H1n2—N2—H2n2	121 (4)	C7—C8—H1c8	119.84
C2—C1—C9	121.8 (3)	C9—C8—H1c8	119.84
C2—C1—H1c1	119.12	C1—C9—C8	122.1 (3)
C9—C1—H1c1	119.12	C1—C9—C10	119.1 (3)
C1—C2—C3	118.9 (3)	C8—C9—C10	118.7 (3)
C1—C2—C11	121.5 (3)	C4—C10—C5	122.6 (3)
C3—C2—C11	119.5 (3)	C4—C10—C9	118.3 (3)
C2—C3—C4	120.8 (3)	C5—C10—C9	119.2 (3)
C2—C3—H1c3	119.59	S1—C11—C2	111.7 (2)
C4—C3—H1c3	119.58	S1—C11—H1c11	109.47
C3—C4—C10	121.1 (3)	S1—C11—H2c11	109.47
C3—C4—H1c4	119.45	C2—C11—H1c11	109.47
C10—C4—H1c4	119.45	C2—C11—H2c11	109.47
C6—C5—C10	120.7 (3)	H1c11—C11—H2c11	107.17
C6—C5—H1c5	119.66	S1—C12—N1	119.8 (3)
C10—C5—H1c5	119.66	S1—C12—N2	118.4 (3)
C5—C6—C7	120.3 (3)	N1—C12—N2	121.8 (3)
			
C9—C1—C2—C3	0.0 (2)	C7—C8—C9—C10	−1.8 (5)
C9—C1—C2—C11	−179.8 (3)	C8—C9—C1—C2	179.4 (3)
C1—C2—C3—C4	0.3 (5)	C10—C9—C1—C2	−0.6 (5)
C11—C2—C3—C4	−179.9 (3)	C4—C10—C9—C1	1.0 (5)
C2—C3—C4—C10	0.0 (3)	C4—C10—C9—C8	−179.0 (3)
C3—C4—C10—C5	178.0 (3)	C5—C10—C9—C1	−177.8 (3)
C3—C4—C10—C9	−0.7 (5)	C5—C10—C9—C8	2.2 (5)
C4—C10—C5—C6	−179.5 (3)	C1—C2—C11—S1	91.8 (3)
C9—C10—C5—C6	−0.8 (5)	C3—C2—C11—S1	−88.0 (3)
C10—C5—C6—C7	−1.1 (5)	C2—C11—S1—C12	−68.1 (3)
C5—C6—C7—C8	1.5 (5)	C11—S1—C12—N1	−68.5 (3)
C6—C7—C8—C9	0.0 (3)	C11—S1—C12—N2	110.7 (3)
C7—C8—C9—C1	178.2 (3)		

### Hydrogen-bond geometry (Å, º)

**Table:**

*D*—H···*A*	*D*—H	H···*A*	*D*···*A*	*D*—H···*A*
N1—H1*n*1···Br1	0.86 (3)	2.54 (3)	3.332 (3)	153 (3)
N1—H2*n*1···Br1^i^	0.86 (2)	2.49 (2)	3.350 (3)	180 (3)
N2—H1*n*2···Br1^ii^	0.860 (19)	2.55 (3)	3.381 (3)	164 (4)
N2—H2*n*2···Br1	0.86 (3)	2.68 (3)	3.434 (3)	147 (3)

Symmetry codes: (i) *x*, *y*−1, *z*; (ii) *x*, −*y*+3/2, *z*−1/2.
